# Dental Exhibitions

**Published:** 1861-01

**Authors:** J. Taft


### DENTAL EXHIBITIONS.

BY J. TAFT.

WHILE reading the protest of the dentists of Upper and Lower Canada against dental exhibitions, some thoughts were suggested in regard to the subject. While we would not for a moment impugn the motives of many who exhibit specimens of dental work at fairs, we do think it competent and proper to hold up the principle and examine it in the light of reason and common sense.

The dental profession is a branch of the medical, which has for its prerogative the relief of human suffering, and ministering to the well-being and comfort of mankind, and it is that, which they who need its aid, should seek, rather than to be lured to it. It is, however, right and desirable that the people, or those who require or may require the aid of a physician, should be familiar with the characteristics and tests of capability and attainments. The manner in which this knowledge shall be communicated is the point in question. The knowledge of what a profession has the power to do is one thing, the knowledge of a man's professed ability is another thing. The special advantages of any particular process or mode is still another. The method of communicating the knowledge of man's ability is the definite point for consideration. The best method of accomplishing this is that which will give those concerned the most accurate knowledge, and anything that would tend to lead them astray is to be deprecated. And now we venture the proposition that in no way can the physician or dentist accomplish this so efficiently, or upon so firm and broad foundation, as by fulfilling in the most perfect manner, the demands of the public and his profession upon him. His own deeds, if accomplished in the clearest light of science, will proclaim the truth in regard to his ability with far more force than a thousand paper declarations, or than a whole bazaar of handicraft specimens. The practice of exhibiting specimens of dental work originated before the profession had made much attainment in science, or at least with those persons, whose scientific and professional attainments were not of the highest order. The object of such exhibitions is to announce or advertise the skill and ability of the exhibitor, and to attract the attention of the public to him, and to his operations.

If such exhibitions were, always a true criterion of the ability and skill of the dentist, we should consider the practice one of very doubtful propriety, for it is simply calling the attention of those interested, in an egotistical way, to himself. That this is true, is proven from the fact, that the name of the maker always accompanies the work exhibited. No one would place specimens of dental work on exhibition, without giving any intimation as to who was the maker. If such specimens were true indications of skill, it would be unprofessional to exhibit them, in the way referred to. The impression designed to be conveyed by such exhibitions, is, that the operator has great skill. A correct idea of skill can never be given by an unapplied piece of woık, however beautiful it may be. The value of a piece of dental work, artificial substitutes, for example, does not depend upon the beauty of its finish, or the symmetry and regularity of its different parts, but in its adaptation to the parts for which it is prepared, and its efficiency in this particular can only be determined, even by the dentist himself, by direct application. It is oftentimes necessary to make plates to which teeth are attached exceedingly irregular and unsightly, and the teeth placed upon the plates quite irregularly, in order to look well in the mouth, or to antagonize properly with other teeth. The true skill of the dentist would be quite as well exhibited by soldering together and polishing two pieces of brass, and fitting on neatly a row of teeth to a board, as by the common specimens of mechanical dentistry exhibited at fairs. The regular and symmetrical arrangement of a set of teeth on a plate, does not give the least indication of the ability of the operator to select and arrange teeth to suit the various countenances, forms and expressions which may be presented. We repeat that such specimens are no indication of the skill of an operator. even in regard to mechanical dentistry; but if it did exhibit his skill perfectly in that department, it shows nothing in regard to his ability in that far more important particular of operative, surgical and medical dentistry. Indeed it is evidence that these departments are undervalued, and of far less importance, in the mind of the exhibitor, than the mechanical. The real intention of dental exhibitions is not to give those concerned a correct knowledge of the resources of the profession, but it is to attract attention to the operator, and to gain business; inasmuch as it does not give a correct knowledge, its tendency is to deception. In regard to gaining business by such methods, the history of every one who has ever attempted it, will prove it a failure. The persons who will be influenced by such exhibitions are of little value as patients, and those who are desirable patients will be repelled by such things. Every discriminating mind will see that there is no true test of skill in such things, and that the tendency is to deceive, and hence they will avoid them. We object again, because such exhibitions are degrading and debasing to any branch of the medical profession, and are so regarded by all truly professional men.

